# Progesterone acts via the progesterone receptor to induce adamts proteases in ovarian cancer cells

**DOI:** 10.1186/s13048-016-0219-x

**Published:** 2016-02-25

**Authors:** Maíra A. Lima, Suély V. da Silva, Vanessa M. Freitas

**Affiliations:** Cell and Developmental Biology Department, Biomedical Sciences Institute (ICB), University of Sao Paulo, Avenida Professor Lineu Prestes, 1524, Biomédicas 1, room 428, São Paulo, SP 05508-000 Brazil

**Keywords:** Ovarian cancer, Extracellular matrix, Matrix metalloproteinases, ADAMTS, Hormones, Androgen receptor, Estrogen receptor and Progesterone receptor, ES-2, NIH-OVCAR-3

## Abstract

**Background:**

Ovarian carcinomas, usually associated with sex hormones dysregulation, are the leading cause of gynecological neoplastic death. In normal ovaries, hormones play a central role in regulating cell proliferation, differentiation, and apoptosis. On the other hand, hormonal alterations also play a variety of roles in cancer. Stimulation by sex hormones potentially affects gene expression, invasiveness, cell growth and angiogenesis. Proteases of the “a disintegrin and metalloproteinase with thrombospondin motifs” (ADAMTS) family are secreted by different cell types and become involved in collagen processing, cleavage of the proteoglycan matrix, and angiogenesis. We evaluated whether sex hormones affect ADAMTS 1 and 4 expression in ovarian cancer cells.

**Methods:**

We analysed mRNA and protein levels in human ovarian tumor cells with different degrees of malignancy, NIH-OVCAR-3 and ES-2, that were treated or not with estrogen, testosterone and progesterone.

**Results:**

Our results suggest that progesterone increases ADAMTS protein and mRNA levels in the lysates from ES-2 cells, and it increases ADAMTS protein in the lysates and conditioned media from NIH-OVCAR-3. Progesterone effects were reversed by RU486 treatment.

**Conclusion:**

We conclude that progesterone acts via the progesterone receptor to modulate ADAMTS 1 and 4 levels in ovarian cancer cell lines.

**Electronic supplementary material:**

The online version of this article (doi:10.1186/s13048-016-0219-x) contains supplementary material, which is available to authorized users.

## Background

Ovarian cancer represents the most lethal malignancy of the female reproductive system [1]. Poor prognosis for women with late-stage disease results in large part from therapy ineffectiveness [2].

In normal ovaries, hormones play a central role in regulating cell proliferation, differentiation, and apoptosis. However, hormonal alterations also play a role in ovarian cancer, affecting gene expression, invasiveness, cell growth, and angiogenesis [3].

Androgens constitute the major pre- and postmenopausal ovarian hormonal product, and serve as required substrates for estrogen synthesis. High androgen levels correlate with increased risk of ovarian cancer development. Moreover, the incidence of ovarian cancer increases after the menopause, when androgens represent the main steroids produced by the ovary [4, 5].

The corpus luteum secretes progesterone, a key component in the regulation of female reproductive tissue growth and development. In the ovary, progesterone plays a central role in regulating ovulation and luteinization via classical receptor-mediated pathways [6]. In ovarian cancer, progesterone might protect against tumor development [7].

Estradiol is predominantly responsible for secondary sexual characteristics in women. Studies have shown that estrogen stimulates growth of ovarian tumor cell lines expressing estrogen receptors (ER) [8]. Epidemiological studies have indicated that estrogen replacement therapy in postmenopausal women may increase ovarian cancer incidence as well as mortality rates [9, 10].

Hormones also affect the tumor microenvironment, composed of soluble factors and extracellular matrix (ECM) molecules. The protease a disintegrin and metalloproteinase with thrombospondin motifs (ADAMTS) is an MMP-related enzyme [11]. This protein has a multi-domain structure and various functions, including collagen processing, cleavage of the proteoglycan matrix [12], cleavage of the von Willebrand factor, as well as roles in inflammation, organogenesis and fertility [13, 14]. These proteases also have anti-angiogenic effects [15–17], and a review of the literature shows that the expression of ADAMTS1 is decreased in different types of cancer, including ovarian cancer [18], what indicates an important role this protease in ovarian health. Furthermore ADAMTS 1 mutations may affect the outcome of ovarian cancers [19].

In the normal ovary, during the LH-induced process of ovulation, the release of a mature oocyte through the surface epithelium requires remodeling of the ovarian ECM. This process is associated with cell-specific expression of numerous proteases [20]. Among these proteins, the normal ovary expresses, for example, ADAMTS 1 [21] and 4 [22]. Sex hormones represent potentially important regulators of ADAMTS expression [23]. However, the correlation between steroid hormones and ADAMTS expression has not been assessed in ovarian cancer models.

In this study, we evaluated the levels of hormone receptors and the effects of testosterone, progesterone and estrogen on the expression of ADAMTS 1 and 4 mRNA and protein in human ovarian tumor cells with different degrees of malignancy including NIH-OVCAR-3 and ES-2.

## Results

### NIH-OVCAR-3 and ES-2 cells expression of estrogen, progesterone and testosterone receptors

**Fig. 1:**
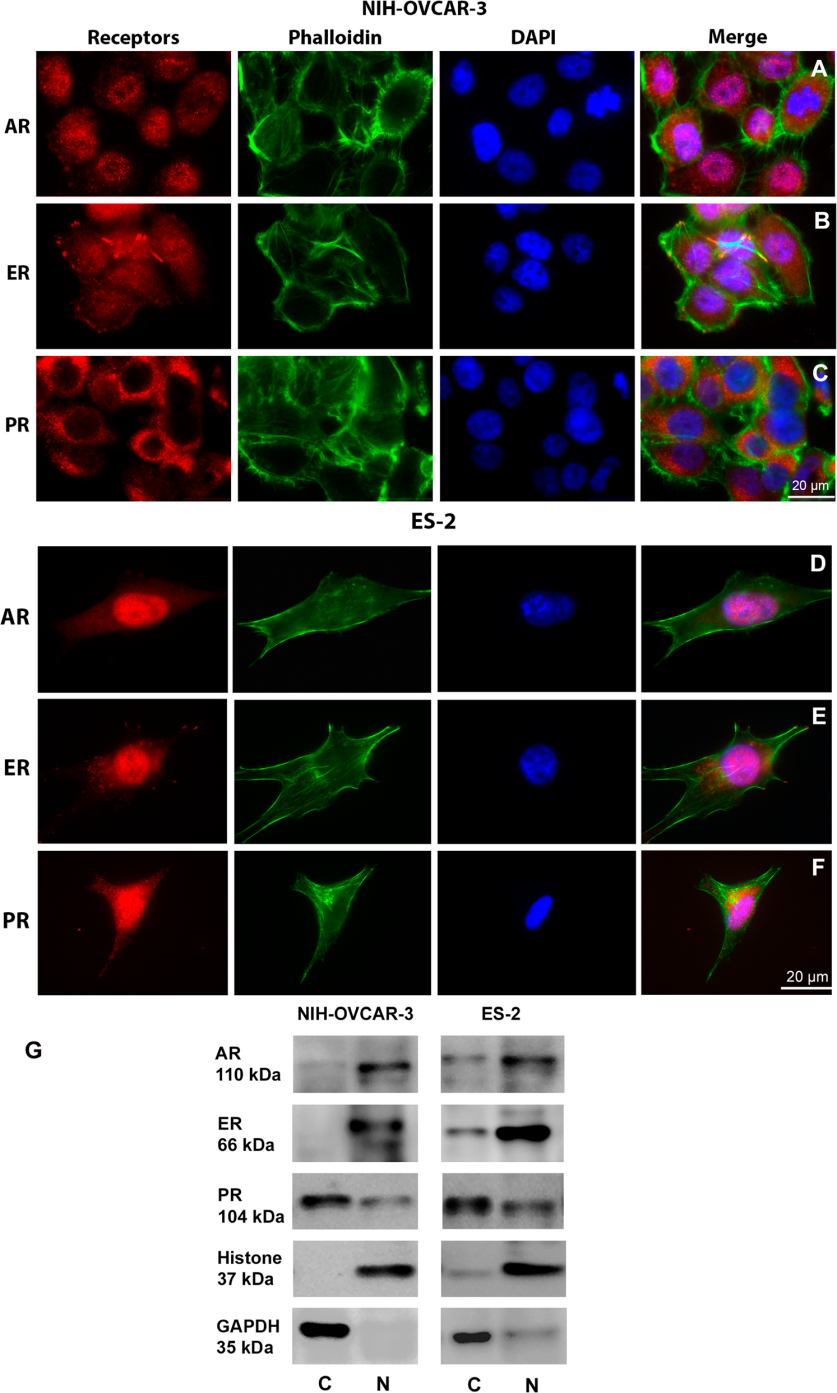
Distribution of AR, ER and PR in NIH-OVCAR-3 and ES-2 cells. AR, ER and PR (red), nuclei (blue) and actin (green). Merge dots in magenta indicate the colocalization of the receptors with the nucleus. In NIH-OVCAR-3 cells **a** Androgen receptor and **b** Estrogen receptor are mostly in the nucleus, but also appears in cytoplasm. **c** Progesterone receptor is mostly found in the cell cytoplasm. In ES-2 cells **d** Androgen receptor and **e** Estrogen receptor are also mainly in the cell nucleus. **f** Progesterone receptor is located in cytoplasm and nucleus. **g** Immunoblot of NIH-OVCAR-3 and ES-2 cell fractions for receptors. In NIH-OVCAR-3 cells ER and AR are present in the nucleus, PR is present in both compartments, but mostly in the cytosolic fraction. ES-2 cells present most of the AR and ER in the nuclear fraction and PR is preferentially in the cytosolic compartment. Scale bar: 20 μm. (C = cytosolic fraction, *N* = nuclear fraction)

Receptors for androgen (AR) and estrogen (ER) were present in the nucleus of the more differentiated cell line, NIH-OVCAR-3, as observed by immunofluorescence, however, they also appear distributed in the cell cytoplasm (Fig. 1a, b). On the other hand, progesterone receptor (PR) was present mostly in the cell cytoplasm (Fig. 1c). Western blot after cell fractionation showed that PR was observed mostly in the cell cytoplasm but also at the nucleus, while ER and AR are present only in the nucleus (Fig. 1g).

In the less differentiated cell line, ES-2, immunofluorescence showed that receptors distribution was similar to NIH-OVCAR-3, AR and ER mostly in the cell nucleus (Fig. 1d, e) but PR was present in the cytoplasm and nucleus (Fig. 1f). Western blot after cell fractionation corroborated immunofluorescence findings, since the same pattern was observed, ER and AR are present mostly in the cell nucleus but also in the cytoplasm and PR present mostly in the cell cytoplasm but a smaller amount in the cell nucleus (Fig. 1g).

### Hormone treatments do not affect cell viability but differentially affect mRNA and protein expression of ADAMTS 1 and 4

**Fig. 2:**
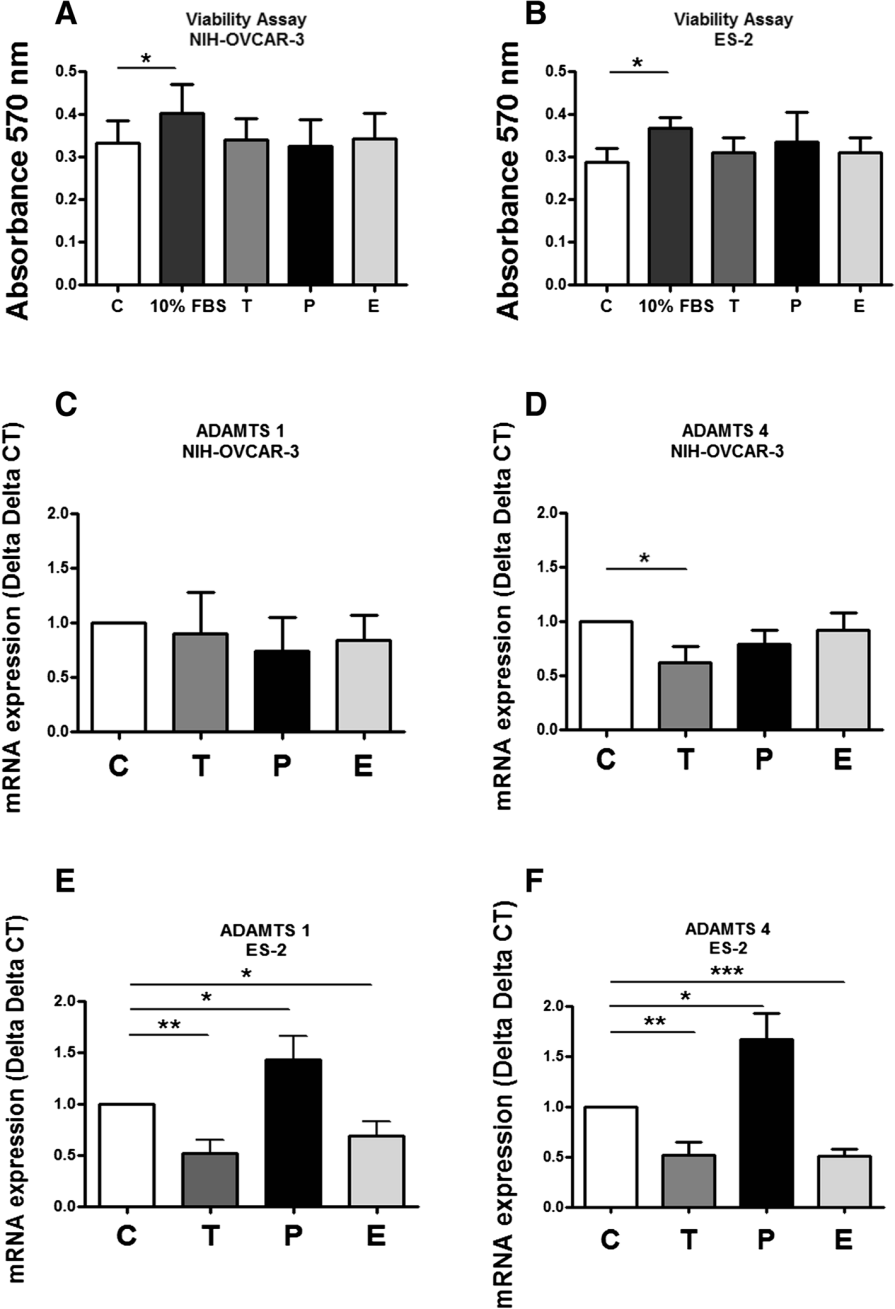
Cell viability and ADAMTS mRNA expression in NIH-OVCAR-3 and ES-2 cell lines, after hormone treatment. **a** and **b** MTT viability assay of NIH-OVCAR-3 and ES-2 cell lines treated with 30 nM estrogen, 1 μM progesterone or 100 nM testosterone for 24 h compared to control. **c** and **d** ADAMTS 1 and 4 mRNA expression assessed by qPCR in the lysates of NIH-OVCAR-3 cells. GAPDH was used as normalizer. **e** and **f** ADAMTS 1 and 4 mRNA expression assessed by qPCR in the cell lysates of ES-2 cells. GAPDH was used as normalizer. Differences among groups were considered statistically significant when *p* ≤ 0.05. Experiments were performed in triplicate

The hormones tested are known to increase proliferation of other cell lines. Neither cell line, NIH-OVCAR-3 or ES-2, reacted to hormone treatments with changes in viability in comparison to the respective serum-free controls (Fig. 2a, b).

Hormone treatment did not significantly alter ADAMTS 1 mRNA expression in the more differentiated cell line, NIH-OVCAR-3 (Fig. 2c). Only testosterone treatment, in comparison to control, induced a significant decrease in ADAMTS 4 gene expression in NIH-OVCAR-3 cells (Fig. 2d). In the less differentiated cell line, ES-2, mRNA levels of ADAMTS 1 and 4 were reduced by estrogen and testosterone treatment in comparison to control (Fig. 2e, f). We also observed an increase in the expression of ADAMTS 1 and 4 mRNA in ES-2 cells treated with progesterone in ES-2 (Fig. 2e, f).

**Fig. 3:**
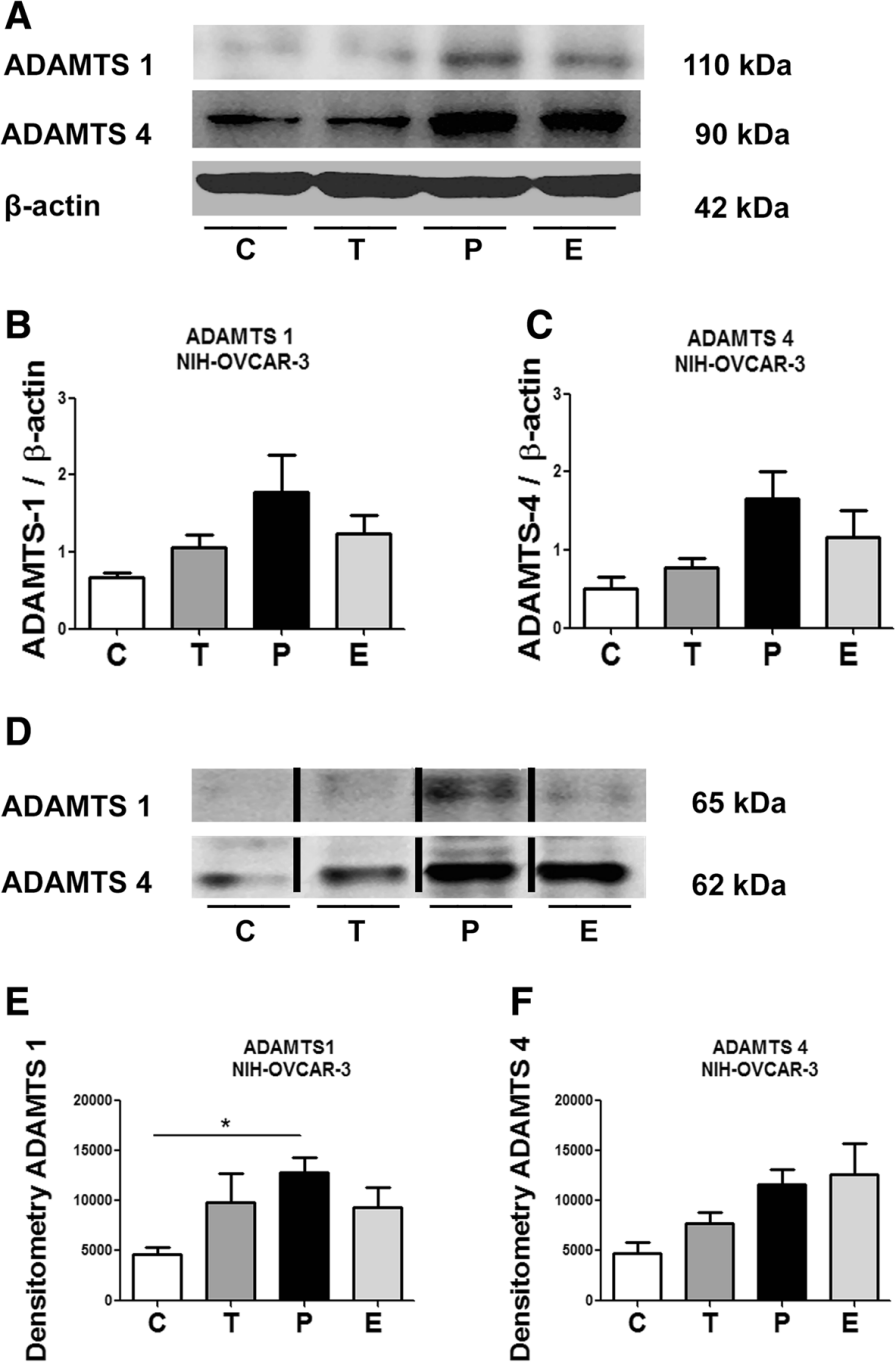
ADAMTS 1 and 4 protein expression of NIH-OVCAR-3 cell lysates and conditioned medium, after hormone treatment. **a** Representative Western blot of ADAMTS 1 and 4 in the lysates of NIH-OVCAR-3 cells treated with 30 nM estrogen (*E*), 1 μM progesterone (*P*), or 100 nM testosterone (*T*), compared to control (*C*). β-actin was used as loading control. **b** and **c** ADAMTS 1 and 4 protein expression in the lysates of NIH-OVCAR-3 cells. **d** Representative Western blot of ADAMTS 1 and 4 in the conditioned medium from NIH-OVCAR-2 cells. **e** and **f** ADAMTS 1 and 4 protein expression in the conditioned medium from NIH-OVCAR-3 cells. Conditioned medium equal sample loading was confirmed by Pounceau S staining (Additional file 1: Fig. S1a). Differences among groups were considered statistically significant when *p* ≤ 0.05. Experiments were performed in triplicate

Protein levels of ADAMTS 1 and 4 presented a tendency to increase when treated by progesterone in NIH-OVCAR-3 cell lysates (Fig. 3a–c). Analysis of the NIH-OVCAR-3 conditioned medium showed that progesterone treatment increased ADAMTS 1 and 4 protein levels in comparison to controls (Fig. 3d–f and S1a).

**Fig. 4:**
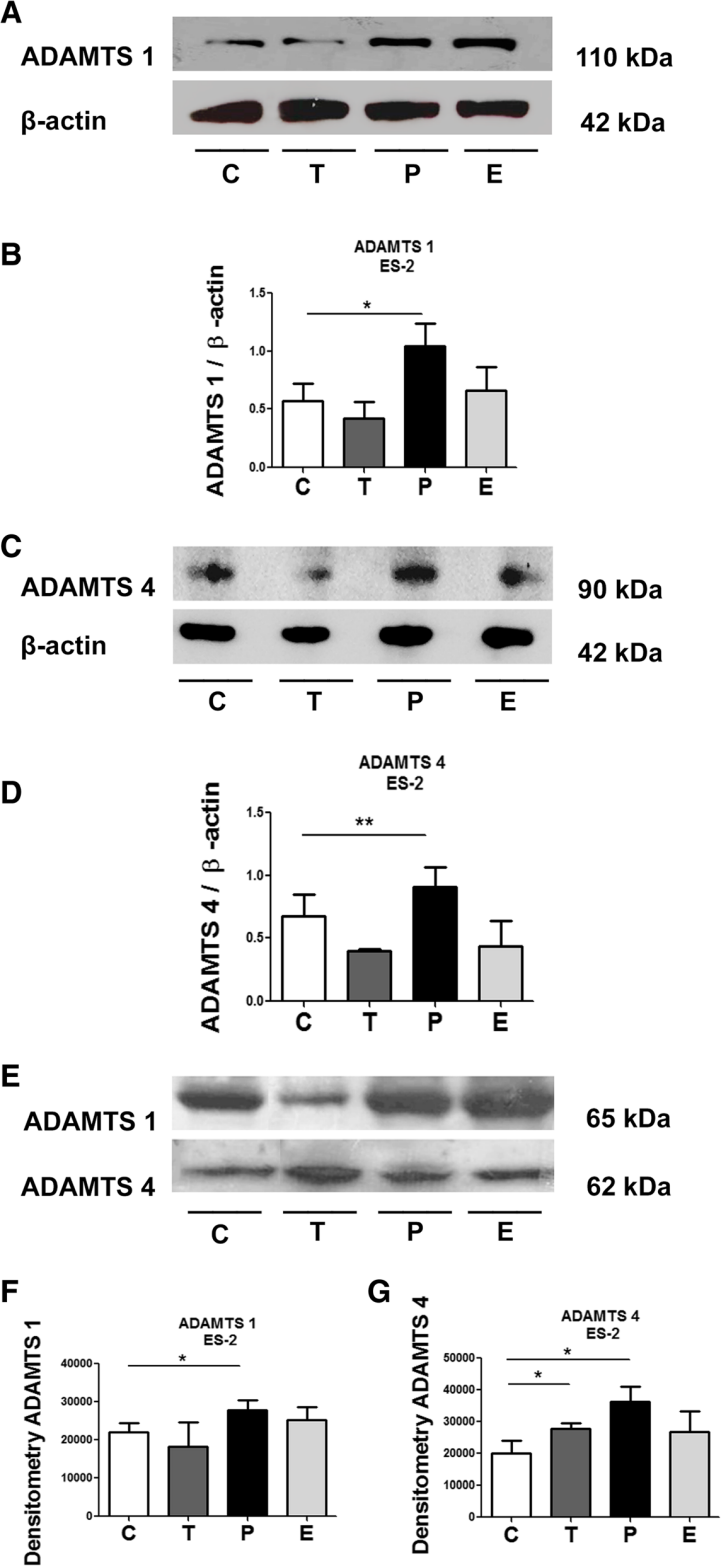
Progesterone induces increase of ADAMTS 1 and 4 in cell lysates and conditioned medium of ES-2 cell. **a** and **c** Representative Western blot of ADAMTS 1 and 4 in the lysates of ES-2 cells treated with 30 nM estrogen (*E*), 1 μM progesterone (*P*) or 100 nM testosterone (*T*), compared to control (*C*). β-actin was used as loading control. **b** and **d** ADAMTS 1 and 4 protein expression in the lysates of ES-2 cells. **e** Representative Western blot of ADAMTS 1 and 4 in the conditioned medium from ES-2 cells. **f** and **g** ADAMTS 1 and 4 protein expression in the conditioned medium from ES-2 cells. Conditioned medium equal sample loading was confirmed by Pounceau S staining (Additional file 1: Fig. S1b). Differences among groups were considered statistically significant when *p* ≤ 0.05. Experiments were performed in triplicate

In ES-2 cells, progesterone induced ADAMTS 1 and 4 protein levels increase (Fig. 4a–d). Hormone treatments had the same effect on ADAMTS 1 and 4 protein levels in ES-2-conditioned media (Fig. 4e–g and Additional file 1: Figure S1b). Addicionally, there was an increase in ADAMTS 4 in the conditioned medium when ES-2 cells were treated by testosterone (Fig. 4g).

### RU486 reverses progesterone mediated induction of ADAMTS 1 and 4 protein expression

To evaluate whether the progesterone induced increase in ADAMTS 1 and 4 protein levels in NIH-OVCAR-3 and ES-2 cells resulted from activation of the progesterone receptor, we conducted a set of experiments in which the progesterone receptor antagonist RU486 was added to the media with progesterone.

**Fig. 5:**
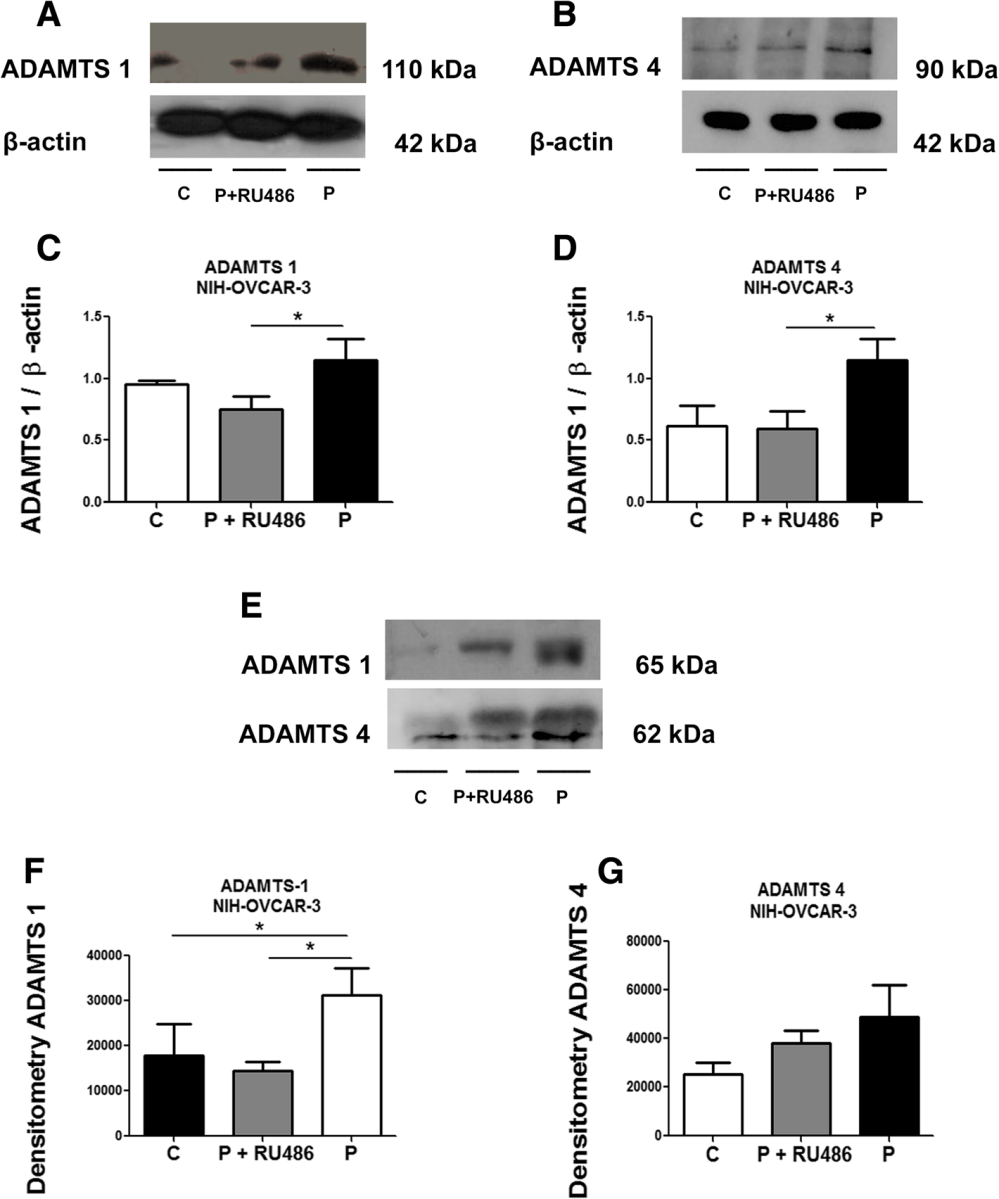
RU486 can rescue the increase of ADAMTS 1 induced by progesterone in NIH-OVCAR-3 conditioned medium. **a** and **b** Representative Western blot of ADAMTS 1 and 4 in the lysates of NIH-OVCAR-3 cells treated with progesterone (*P*) or progesterone and RU486 (P + RU486) compared to control (*C*). **c** and **d** ADAMTS 1 and 4 protein expression in the lysate of NIH-OVCAR-3 cells. **e** Representative Western blot of ADAMTS 1 and 4 in the conditioned medium from NIH-OVCAR-3 cells. **f** and **g** ADAMTS 1 and 4 protein expression in conditioned medium of NIH-OVCAR-3. Conditioned medium equal sample loading was confirmed by Pounceau S staining (Additional file 1: Fig. S1c). Differences among groups were considered statistically significant when *p* ≤ 0.05. Experiments were performed in triplicate

Co-treatment with RU486 showed decrease of progesterone-mediated induction of ADAMTS 1 (Fig. 5a, c) and 4 (Fig. 5b, d) in the cell lysate of NIH-OVCAR-3 cells. The progesterone receptor antagonist also reversed the effects of progesterone on ADAMTS 1 protein levels in NIH-OVCAR-3 conditioned media (Fig. 5e, f and Additional file 1: Figure S1c), whereas no significant effects were observed regarding ADAMTS 4 (Fig. 5e, g).

**Fig. 6:**
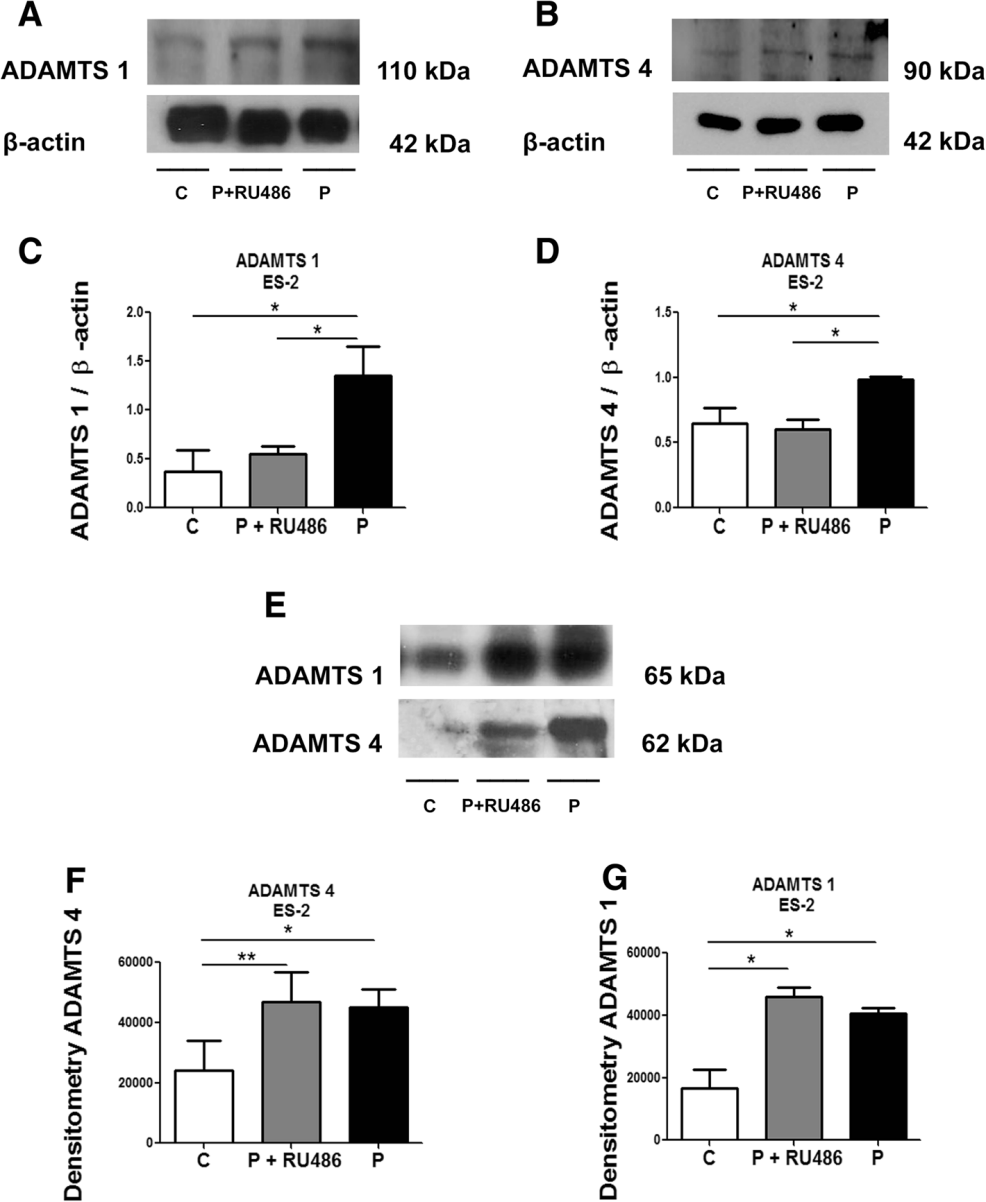
RU486 can rescue the increase of ADAMTS 1 and 4 induced by progesterone in ES-2 cell lysates but not in the conditioned medium. **a** and **b** Representative Western blot of ADAMTS 1 and 4 in the lysates of ES-2 cells treated with progesterone (*P*) or progesterone and RU486 (P + RU486) compared to control (*C*). **c** and **d** ADAMTS 1 and 4 protein expression in the lysates of ES-2 cells. **e** Representative Western blot of ADAMTS 1 and 4 in the conditioned medium from ES-2 cells treated as above. **f** and **g** ADAMTS 1 and 4 protein expression in the conditioned medium from ES-2 cells. Conditioned medium equal sample loading was confirmed by Pounceau S staining (Additional file 1: Fig. S1d). Differences among groups were considered statistically significant when *p* ≤ 0.05. Experiments were performed in triplicate

RU486 treatment reversed the progesterone effect on ADAMTS 1 and 4 in ES-2 cells lysates (Fig. 6a–d). In ES-2-conditioned media, progesterone treatment resulted in an increase of expression of ADAMTS 1 and 4 compared to control (Fig. 6e–g and Additional file 1: Figure S1d). However, treatment with the PR antagonist RU486 was ineffective in reversing these effects (Fig. 6e–g and Additional file 1: Figure S1d).

## Discussion

Proteolytic modification of cell-surface proteins and extracellular matrices represents a fundamental step for a diverse array of biological and pathological processes, including embryogenesis, wound healing, and cancer metastasis [20]. In this study we observed ADAMTS 1, 65 kDa (fragmented or processed) in the cells conditioned medium and ADAMTS 1, 110 kDa (full-length or unprocessed) was detected in the cell lysate of both cell lines studied. Full-length ADAMTS 1 has pro-tumor activity, whereas the fragmented protein has anti-tumor activity. Also, full length ADAMTS 1 increases tumor cell proliferation rates and accelerates tumor development through the activity of its metalloproteinase domain [24]. With regard to epigenetic mechanisms in ovarian carcinomas, ADAMTS 1 gene has been described as not methylated [25]. More recently, a comprehensive study showed that members of the ADAMTS family, including ADAMTS 1, present somatic mutations that are associated to chemotherapy outcome in ovarian cancer patients [19]. Concerning ADAMTS 4, we observed that ADAMTS 4, 90 kDa (full-length or unprocessed) in the cell lysate and ADAMTS 4, 62 kDa (fragmented or processed) is detected in the conditioned medium of both ovarian cancer cell lines studied. Other group has demonstrated that full-length ADAMTS 4 promoted melanoma tumor growth. In contrast, the C-terminal ancillary regions and the full-length protein lacking catalytic activity inhibited tumor growth [26]. Thus, ADAMTS 4 behaves similarly to ADAMTS 1 in that it has both pro-and anti-tumorigenic activities promoted depending on its form. Here, we detected different forms of ADAMTS 1 and ADAMTS 4 depending on cell localization. As expected for an extracellular protease the processed or activated form was found secreted in the cell’s conditioned medium.

MTT revealed that hormones, in the concentration used here, did not alter cell viability of NIH-OVCAR-3 or ES-2 cell lines, when compared to serum-free control group. Thus, changes in the levels of gene and protein expression of proteases are unrelated to the increase or decrease in cell number induced by hormones. The ES-2 cell line, when treated with estrogen or testosterone, showed decreased mRNA levels of ADAMTS 1 and 4, while progesterone treatment increased the mRNA levels in both cell lines. At the protein level, progesterone increased ADAMTS 1 and 4 expression in ES-2 cell lysates and conditioned medium.

Regarding the cell line NIH-OVCAR-3, we observed that testosterone treatment reduced the mRNA expression of ADAMTS 4. Western blot analysis indicated that progesterone treatment induced increased of protein levels of ADAMTS 1 in the conditioned medium. Despite the increase in ADAMTS 1 protein expression induced by progesterone, this hormone was not able to induce an increase in ADAMTS 1 gene expression in the same NIH-OVCAR-3 cell line. Northern blot analysis showed that ADAMTS 1 gene expression reaches its maximum levels after 12 h post hormone stimulation and this expression declined significantly during early luteal formation [21]. In this work we analysed mRNA expression after 24 h post-treatment and the ADAMTS 1 mRNA levels could be decreased at this point. We also observed that RU486 reversed the effects of progesterone on the levels of ADAMTS 1 in the lysates from both cell lines and ADAMTS 4 in ES-2 cells, which further suggests that progesterone acts directly through its receptor to increase the expression of this proteases in ovarian cancer cell lines.

In the present study we observe that both NIH-OVCAR-3 and ES-2 cells express PR, mainly in the cytoplasm but also in the nuclei. Considering that both cell lines express the same PR pattern, we speculate that the absence of RU486 effect in decreasing ADAMTS 1 and 4 from the conditioned medium stimulated by progesterone could be related to the secretory pathway of these proteases in ES-2 cell line.

Previous studies have shown that progesterone is the most potent regulator of ADAMTS gene expression [23]. Progesterone elicits its physiological effects especially through the activation of the progesterone receptor (PR) [27]. Thus, progesterone-receptor-null mice fail to ovulate and express markedly reduced levels of ADAMTS 1 [23]. Physiologically the progesterone receptor (PR) gene is activated by the action of luteinizing hormone (LH) in the ovaries. Progesterone binds to its receptor in granulosa cells, resulting in increased ADAMTS 1 [28, 29]. The ADAMTS 1 gene does not have a progesterone responsive element. Thus, PR-mediated induction of the ADAMTS gene seems to occur indirectly through interactions of PR with the transcriptional regulators C/EBPβ, NF1-like factor, and Sp1/3. In this sense, PR plays the role of an ADAMTS 1 coregulator in granulosa cells [28]. The protective effects of progesterone could be mediated by the nuclear progesterone receptor (n-Pr), which is gradually lost with increasing ovarian cancer malignancy [30]. Here, we observe that besides the differences in cell morphology and behavior, NIH-OVCAR-3 and ES-2 have similar PR expression and localization under the same circumstances (without hormone treatment and phenol red) and the effects of progesterone were similar in both cell lines.

ADAMTS 1 and 4 have different functions depending on their molecular conformation [24, 26] and they can bind the vascular endothelial growth factor (VEGF). In the ECM, ADAMTS 1 and 4 might sequester VEGF, preventing it from binding to its cellular receptor, and ultimately leading to a decrease in cell migration and invasion rates [26, 31]. Progesterone may exert part of its protective effects against ovarian cancer by increasing ADAMTS 1 and 4, which in turn would decrease cell migration and invasion. Further studies should focus on this hypothesis.

## Conclusions

We conclude that progesterone acts via progesterone receptor to regulate ADAMTS 1 production and secretion by differentiated ovarian cancer cells (NIH-OVCAR-3) and in less differentiated cancer cells (ES-2), progesterone receptor inhibitor is able to decrease ADAMTS 1 and 4 in the cell lysate but not in the conditioned medium of ES-2 cells.

## Methods

### Cell lines

The cell line NIH-OVCAR-3 is derived from an ovarian adenocarcinoma and has epithelioid morphology. The cell line ES-2 was developed from a clear cell carcinoma of the ovary and has a fusiform morphology. Cells were obtained from the Cell Bank of Rio de Janeiro. ES-2 and NIH-OVCAR-3 cells were cultured in Dulbecco’s Modified Eagle’s Medium-F12 (DMEM-F12, Sigma Chemical Co, St. Louis, MO, USA) supplemented with 10 % fetal bovine serum (FBS; Cultilab, Campinas, SP, Brazil). Cells were maintained in 75 cm^2^ flasks in a humidified atmosphere of 5 % CO_2_ at 37 °C.

### Immunofluorescence

Cells grown on glass coverslips was fixed in 4 % paraformaldehyde in phosphate-buffered saline (PBS) for 10 min, rinsed and permeabilized with 0,5 % Triton X-100 (Sigma) in PBS for 10 min, rinsed and blocked with Normal Goat Serum (10 %) for 1 h, followed by incubation with primary antibody rabbit polyclonal against the N-terminus of AR (N20, Santa Cruz Biotech, Santa Cruz, CA) 1:500, rabbit polyclonal against the C-terminus of PR (C19, Santa Cruz Biotech, Santa Cruz, CA) 1:500 or rabbit polyclonal that recognizes the N-terminus of ER (H184, Santa Cruz Biotech, Santa Cruz, CA) 1:500 overnight, followed by labeling with anti-rabbit Alexa 568 (Invitrogen). After that, cells were incubated with Alexa Fluor 488-phalloidin (Invitrogen). Samples were mounted in Pro Long with DAPI (Invitrogen).

### Hormone treatment

NIH-OVCAR-3 and ES-2 cells were plated in 60-mm^2^ tissue culture dishes (Corning, New York, USA) at a density of 3x10^6^ cells per dish for Western Blot analyses of total lysate and conditioned medium. Cells were plated in 30-mm^2^ tissue culture dishes (Corning, New York, USA) at a density of 1x10^6^ cells per dish for RNA extraction and qPCR analysis. In both cases, cells were grown to 70 % of confluence, then washed with PBS and cultured in phenol red-free Dulbecco’s Modified Eagle’s Medium/F12 (DMEM-F12, Sigma), supplemented with 10 % charcoal-stripped fetal bovine serum for 24 h. After this period, cells were treated with estrogen, progesterone and testosterone (Sigma) in phenol red-free DMEM/F12 at the concentrations of 30nM, 1 μM, and 100 nM, respectively, for 24 h. A group of NIH-OVCAR-3 and ES-2 cells treated with progesterone was also cultivated in the presence of the progesterone inhibitor RU486 (7 μM). Ovary cells lines cultivated without the addition of hormones in phenol red-free DMEM/F-12 served as controls. Treated and control cells were subjected to qPCR and immunoblot analyses, to determine ADAMTS 1 and 4 mRNA and protein levels. All experiments were performed in triplicate.

### MTT cellular viability assay

After hormonal treatment for 24 h, the MTT assay was performed. In summary, cell culture supernatants were removed and a solution was added to each plate containing 100 μl of fresh medium DMEM F12 with 10 μl of an MTT solution (Calbiochem, Darmstadt, Germany) of 5 mg/mL in PBS, resulting in a final MTT concentration of 0.5 mg/ml. Cells were maintained in the incubator for 3 h until blue crystals were formed. At this point, DMSO (100 μl) was added to dissolve the crystals, the plate was homogenized, and absorbance reading was performed at 570 nm in a spectrophotometer.

### Real-time PCR (qPCR)

Total RNA was extracted with the Magna Bead Total RNA kit (Invitrogen, USA) following the manufacturer’s recommendations. Ten micrograms of total RNA, previously treated with DNase, were reverse transcribed using a High Capacity cDNA Archive Kit (Applied Biosystems, Carlsbad, CA, USA). qPCR was performed using an Applied Biosystems 7500 Real-Time PCR System, and each cDNA sample was analysed in duplicate. The PCR reactions were carried out in a total volume of 25 μl according to the manufacturer’s instructions for the SYBR Green PCR Core reagent (Invitrogen, USA). The following PCR primers were used: ADAMTS 1 forward, 5’-TGTGGTGTTTGCGGGGGAAATG-3’ and reverse, 5’- TCGATGTTGGTGGCTCCAGTT-3’; ADAMTS 4 forward, 5’ TCAGCCTTCACTGCTGCTCAT-3’ and reverse 5’-GCCCATTCAAACTGATGCATG 3’; and glyceraldehyde-3-phosphate dehydrogenase (GAPDH) forward, 5’-CCTCCAAAATCAAGTGGGGC G-3’ and reverse, 5’-GGGGCAGAGATGATGACCCTT-3’. As a result, the relative gene expression was normalized, with GAPDH expression serving as the internal control. Results were expressed as the n-fold difference in target gene expression relative to the expression of the GAPDH gene and the reference sample. The relative expression was calculated using the 2^-ΔΔCT^ method [32].

### Cell fractionation

Nuclear–cytoplasmic fractionation was performed using the NE-PER Nuclear and Cytoplasmic Extraction Reagents kit (Thermo Fisher Scientific) according to the manufacturer’s protocol, and then quantified (BCA kit, Pierce).

### Western blot

Western blots were carried out in order to compare ADAMTS 1 and 4 levels in ES-2 and NIH-OVCAR-3 cell total lysates and conditioned medium and to analyse steroid hormone receptors from fractions described above. For the cell lysate protein preparation, cells were lysed in RIPA buffer (150 mM NaCl, 1.0 % NP-40, 0.5 % deoxycholate, 0.1 % SDS, 50 mM Tris pH 8.0) containing a protease inhibitor cocktail (Sigma). After centrifugation (10,000 g) for 10 min at 4 °C, the supernatants were recovered and quantified (BCA kit, Pierce). Protein from the conditioned medium (1 mL) was obtained by ethanol precipitation. For SDS-PAGE, 30 μg of protein from lysate and all precipitate from conditioned medium were loaded per well and separated in 10 % polyacrylamide gel (prepared with 1.5 M Tris–HCl, 10 % SDS, 30 % bis-acrylamide, 10 % ammonium persulfate, and TEMED). Gel contents were transferred to a Hybond ECL nitrocellulose membrane (Amersham), which was then blocked with TBS containing 5 % non-fat milk overnight at 4 °C. After a wash in TBS with 0.05 % Tween 20 (TBST), the membranes were probed with antibodies against ADAMTS 1 (1:1000, Millipore MAB 1810), ADAMTS 4 (1:2000, Abcam ab84792), AR (1:1000, N20, Santa Cruz Biotech), ER (1:1000, H184, Santa Cruz Biotech ), PR (1:1000, C19, Santa Cruz Biotech), Histone (1:4000, 05-457, Millipore), GAPDH (1:4000, 9484, Abcam) or β-actin (1:2000, Sigma). The ECL protocol was used to detect proteins on the membrane.

### Statistical analysis

Data were analysed with the Graph Pad Prism 5 software (Graph Pad Software, Inc., San Diego, CA, USA), and statistical significance was obtained using the One-way Anova or *T* test.

### Ethics approval

Not applicable.

### Consent for publication

Not applicable.

### Endnote

*“This manuscript was reviewed by a professional science editor and by a native English-speaking copy editor to improve readability”.*

## Additional file

Additional file 1: Figure S1. Pouceau staining shows protein loading for the conditioned medium samples from NIH-OVCAR-3 cells (a, c) and ES-2 cells (b, d). (TIF 24541 kb)

## Abbreviations

ADAMTS: a disintegrin and metalloproteinase with thrombospondin motifs
E: estrogen
P: progesterone
T: testosterone
AR: androgen receptor
ER: estrogen receptor
PR: progesterone receptor
MTT: 3-(4,5-dimethylthiazol-2-yl)-2,5-diphenyltetrazolium bromide
GAPDH: glyceraldehyde-3-phosphate dehydrogenase
