# Supplementary figures and images for: Progesterone acts via the progesterone receptor to induce adamts proteases in ovarian cancer cells
Source: J Ovarian Res. 2016 Feb 25;9:9. doi: 10.1186/s13048-016-0219-x (PMC4766681; doi:10.1186/s13048-016-0219-x)

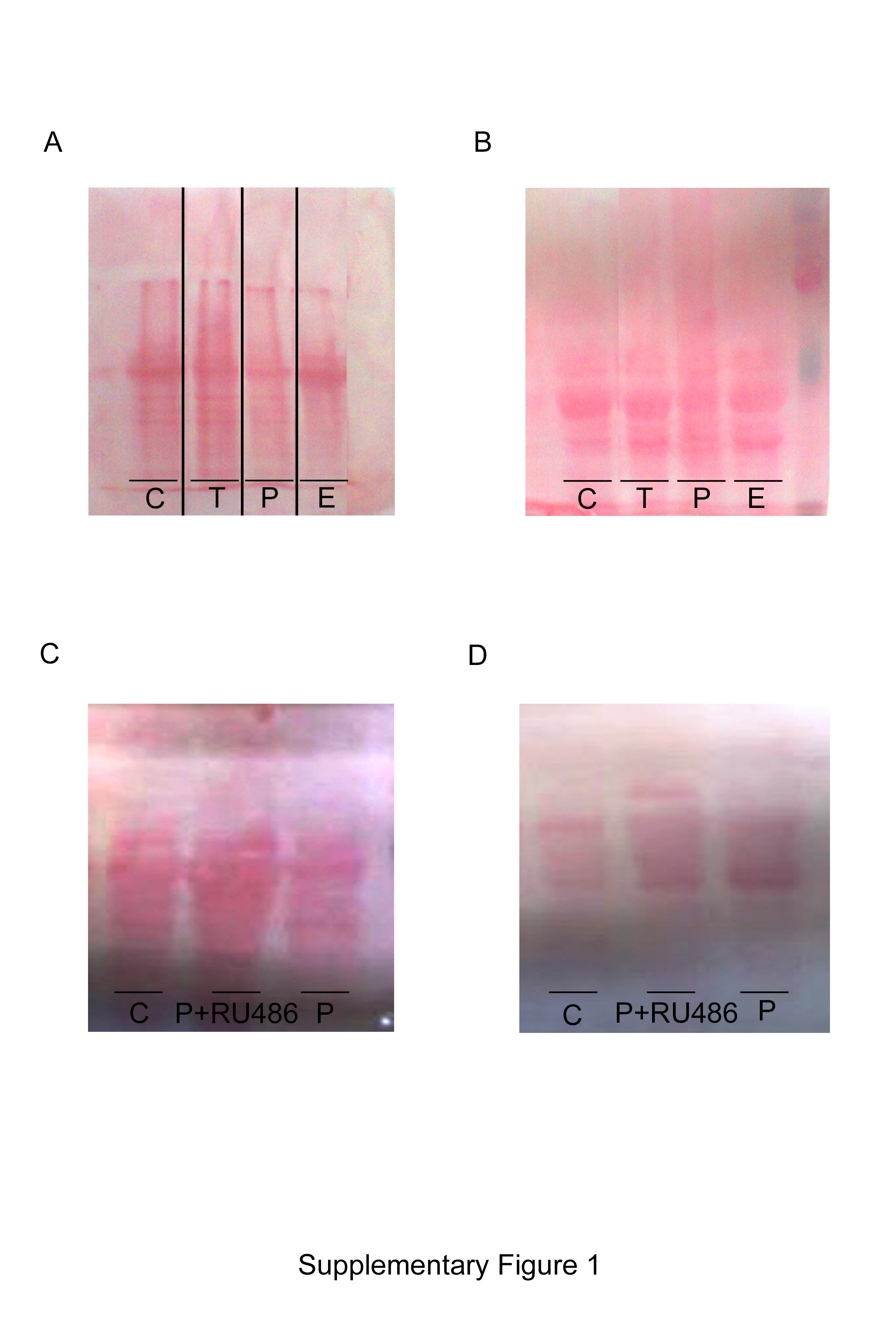

Supplement: Additional file 1: Figure S1. — Pouceau staining shows protein loading for the conditioned medium samples from NIH-OVCAR-3 cells (a, c) and ES-2 cells (b, d). (TIF 24541 kb) [file 13048_2016_219_MOESM1_ESM.tif]
